# Harnessing a clinician-led governance model to overcome healthcare tribalism and drive innovation: a case study of Northumbria NHS Foundation Trust

**DOI:** 10.1108/JHOM-05-2022-0157

**Published:** 2022-12-19

**Authors:** Nancy S. Bolous, Dylan E. Graetz, Hutan Ashrafian, James Barlow, Nickhill Bhakta, Viknesh Sounderajah, Barrie Dowdeswell

**Affiliations:** Department of Global Pediatric Medicine , St Jude Children’s Research Hospital , Memphis, Tennessee, USA; Institute of Global Health Innovation, Imperial College London , London, UK; Department of Surgery and Cancer, Imperial College London , London, UK; Business School, Imperial College London , London, UK; Management Center Innsbruck, Internationale Hochschule GmbH , Innsbruck, Austria

**Keywords:** Healthcare tribalism, Governance reform, Clinician-led managerial model

## Abstract

**Purpose:**

Healthcare tribalism refers to the phenomenon through which different groups in a healthcare setting strictly adhere to their profession-based silo, within which they exhibit stereotypical behaviours. In turn, this can lead to deleterious downstream effects upon productivity and care delivered to patients. This study highlights a clinician-led governance model, implemented at a National Health Service (NHS) trust, to investigate whether it successfully overcame tribalism and helped drive innovation.

**Design/methodology/approach:**

This was a convergent mixed-methods study including qualitative and quantitative data collected in parallel. Qualitative data included 27 semi-structured interviews with representatives from four professional groups. Quantitative data were collected through a verbally administered survey and scored on a 10-point scale.

**Findings:**

The trust arranged its services under five autonomous business units, with a clinician and a manager sharing the leadership role at each unit. According to interviewees replies, this equivalent authority was cascaded down and enabled breaking down professional siloes, which in turn aided in the adoption of an innovative clinical model restructure.

**Practical implications:**

This study contributes to the literature by characterizing a real-world example in which healthcare tribalism was mitigated while reflecting on the advantages yielded as a result.

**Originality/value:**

Previous studies from all over the world identified major differences in the perspectives of different healthcare professional groups. In the United Kingdom, clinicians largely felt cut off from decision-making and dissatisfied with their managerial role. The study findings explain a governance model that allowed harmony and inclusion of different professions. Given the long-standing strains on healthcare systems worldwide, stakeholders can leverage the study findings for guidance in developing and implementing innovative managerial approaches.

## 1. Introduction

Tribalism is defined as *“loyalty to a tribe or other social group, especially when combined with strong negative feelings for people outside the group* ” ( Mannix and Nagler, 2017 ). Throughout human evolution, ingroup loyalties favoured survival and thus natural selection sculpted human minds to be tribalistic, leading to concomitant cognitive biases ( Clark *et al.* , 2019 ). In the healthcare sphere, professional tribalism is a well-established phenomenon ( Davies and Mannion, 2013 ). Despite the shared goal of patient well-being, health care is often provided by siloed working groups, namely, doctors, nurses, allied health professionals (AHPs) and lay managers, all of whom often exhibit discordant attitudes towards each other's working roles ( Degeling *et al.* , 2003 ; Braithwaite *et al.* , 2016 ; Storkholm *et al.* , 2017 ).

The reasons behind these cognitive biases range widely and encompass deeply rooted challenges such as historical gender-restricted roles, educational approach, psychological barriers and organizational distribution ( Braithwaite *et al.* , 2016 ). Historically, professional roles in the healthcare sphere were segregated based on genders, where males assumed the dominant positions as doctors, while females were mostly limited to the more submissive role of nurses. Although society more generally has taken significant steps away from gender stereotyping, the healthcare sector has not fully eliminated the sequelae of the societal malady ( Braithwaite *et al.* , 2016 ). Educational approach poses another obstacle; while modern medical training pays a lot of attention to doctor–patient relationship, less focus is given to ensuring that different healthcare professions can communicate with each other efficiently ( Weller *et al.* , 2014 ). Social identity theory has also been used to explain the ingrained mindsets of different healthcare professionals ( Burford, 2012 ). The theory suggests that establishing professional identity is a process of categorization where a person is conditioned to exhibit the behaviour that people within the group and outside the group expect of him/her. Although authors view development of professional identity as fundamental to professionalism, derogatory stereotyping and generalization of other groups could arise ( Burford, 2012 ; Weller, 2012 ). Additionally, environmental barriers such as geographic distribution, conflicting schedules and incompatible software interfaces exacerbate the disconnection between different healthcare professions ( Weller *et al.* , 2014 ).

All these factors combined result in each professional group prioritizing different aspects and having conflicting perspectives and expectations. Previous research found that physicians often gravitated towards individualism, with autonomy in determining treatment regimens and a focus on health outcomes, nurses were biased towards systemization of work and an emphasis on patient experience and lay managers showed a strong focus on systemizing processes and prioritizing financial reality and accountability ( Degeling *et al.* , 2003 ; Storkholm *et al.* , 2017 ). These conflicting positions have posed a serious impediment to uniformly approaching patient care on a multi-disciplinary basis and were especially exacerbated during any pursuit of change, such as organizational reform ( Degeling *et al.* , 2003 ). For example, physicians, in particular, have been described as defensive and passive during reform processes due to perceived threats to their professional integrity or financial demand ( Dellve *et al.* , 2018 ). Interestingly, clinicians (physicians, nurses and AHPs), when placed in management roles, reported that they largely felt cut off from decision-making and dissatisfied with their managerial role and its influence ( Davies *et al.* , 2003 ; Giordano, 2010 ).

In the United Kingdom's (UK) National Health Service (NHS), a unique set of circumstances add to its governance complexity. The NHS was established in the aftermath of the second world war with the purpose of universally serving every patient for free at the point of delivery while being paid for by central government funding ( Grosios *et al.* , 2010 ). Despite the political changes over the years, the vision and mission of the NHS has not changed ( Grosios *et al.* , 2010 ). Today, the NHS is the world's largest publicly funded health service and one of the world's largest employers ( TheKing'sFund, 2022 ). The sheer size of the NHS and the fact that it is scrutinized by the public and politicians imposes unparallelled expectations and pressure on those holding senior management positions. For clinicians, risks of taking on such a position include being confronted with underlying systemic problems that they often do not have the power to solve, being distanced from their clinical practice and losing its security and risking public humiliation and reputational ruin, all of this for similar or lower salaries than what they earn as clinicians ( Vize, 2015 ).

These reasons could explain why only 58% of NHS managers had any sort of clinical degree, compared to 64% in France, 71% in Germany, 74% in the United States and 93% in Sweden ( Vize, 2015 ). Another study estimated that clinicians comprised only a quarter of the executive board members, of which physicians represented 14% and nurses and AHPs together accounted for 12% ( Veronesi *et al.* , 2013 ). Health systems other than the NHS have shown that a greater number of clinicians in senior management roles contributed to an improvement in health system key performance indicators, such as higher quality ratings of service providers, lower morbidity rates and increased patient satisfaction ( Lega *et al.* , 2013 ). Moreover, breaking down professional silos by engaging different professional groups has been demonstrated as essential to overcome antagonism in health service modernization when responding to reform initiatives ( Degeling *et al.* , 2003 ; Dellve *et al.* , 2018 ).

The Northumbria Healthcare NHS Foundation Trust (NHCT) in the United Kingdom provided a case study of the introduction of an innovative governance structure and its role in mitigating tribalism. The trust was facing unique geographic and demographic challenges which incentivized it to adapt its clinical model in 2015, to ensure accessibility to seven-day specialist service. The process of organizational change proceeded seamlessly and was noted by the Care Quality Commission (CQC), the independent regulator of health and social care in England in their inspection report published in 2016 *“Inspirational leadership and strong clinical engagement had ensured that this change had been managed extremely well and effectively”* ( Commission, 2016 ).

The objective of this study was to describe the clinician-led governance model adopted by the trust and employ theory-generating methodology to explore whether this approach successfully overcame tribal barriers and whether overcoming the tribal barriers potentially contributed to facilitating the process of the clinical model adaptation implemented in 2015.

## 2. Methodology

### 2.1 Study design

The study followed a convergent mixed-methods approach in which qualitative and quantitative data were collected in parallel via interviews, analyzed separately, then synthesized ( Robson and McCartan, 2016 ). The purpose of this design was to gather complementary data enabling the qualitative component to give context and depth to the quantitative findings ( Creswell and Clark, 2017 ). Data collection occurred between May and August 2016 and was carried out by the first author (NSB) to guarantee consistency.

### 2.2 Setting

An NHS trust is an organizational unit within the NHS system (primarily hospitals) serving either a geographical area or a specialized function. NHCT is located in the Northeast of England and covers one of the largest geographical areas of any NHS organization, spanning a catchment area of over 2,500 square miles and a population of approximately 500,000 people. Moreover, it serves a diverse population group split between a widespread rural area and a concentrated heavily urbanized sector comprising former mining and shipbuilding communities with high levels of social deprivation. This disparity is set within the context of common demographic factors such as ageing and increased prevalence of chronic diseases. In addition, severe financial austerity measures such as budget cuts implemented after the 2008–2009 economic crisis had reduced funding income thereby exacerbating the problem and widening the gap of unmet needs and inequities in medical care ( Roberts *et al.* , 2010 ).

Prior to June 2015, the trust comprised 10 medical facilities: three main district general hospitals, six community hospitals, and one dedicated outpatient facility. Given its large geographic catchment area, emergency services were located at the three main hospitals to provide easy access, however this resulted in costly and inefficient duplication of services especially during the weekends when staffing levels were also overstretched. This resulted in elevated mortality over the weekend which is not uncommon. This issue has been identified by previous research and titled ‘The Weekend Effect’ ( Freemantle *et al.* , 2012 ; Wise, 2012 ; Underwood *et al.* , 2019 ; Tolvi *et al.* , 2020 ). To address these issues, the trust adapted its clinical model in June 2015 by opening a purpose designed hospital: the Northumbria Specialty Emergency Care Hospital (NSECH) at Cramlington, central to the main area of population but also adjacent to main countywide access roads. The NSECH was developed as a stand-alone hospital, which centralized all emergency care services at one site, serving the entire catchment population of the trust. This was the first instance in the NHS of a new emergency service hospital developed discrete from a host acute general hospital.

Under the new arrangement, the three district general hospitals' role changed towards a step-down level of care for urgent but not emergency care needs. Each of the general hospitals therefore developed Urgent Treatment Centres (UTC) run predominately by nurse practitioners, general practitioners and AHPs where patients could walk-in for non-emergency conditions. Most patients attending the sub-acute base hospitals/UTCs were seen and treated there, however, patients with life threatening conditions who attended without realizing their critical state were stabilized and transferred to NSECH. Similarly, after initial management, emergency patients who had been stabilized in NSECH could then be transferred after initial management back to the sub-acute base hospital/UTC closest to their residence. Moreover, these hospitals were also able to develop a wider range of elective care and become local hubs for improving the integration of care between acute services and primary care. Further details on the clinical model adaptation are beyond the scope of this paper. Nonetheless, it is important to note that planning and implementing the change went smoothly as noted above ( Commission, 2016 ).

### 2.3 Participants

A purposive sampling approach was adopted to recruit interviewees representing different professional roles; the sample consisted of physicians, lay managers, nurses, and AHPs ( Palinkas *et al.* , 2015 ). For inclusion, participants must have worked with the trust for at least two years. Most participants held managerial positions; however, at least one non-managerial professional from each category was interviewed. Additionally, attention was paid to gender representation. The Chief Executive Officer at the time was asked to recommend candidates who fit the inclusion criteria. After 27 interviews —including pilot interviews— thematic saturation was reached, and no further interviews were pursued ( Hennink *et al.* , 2017 ).

### 2.4 Consent and ethical approval

An Electronic Consent form was sent by email prior to the interview to make sure that interviewees understood the scope of the study and were aware that the interview was being recorded. On the day of the interview, a hard copy of the consent form was signed by each participant prior to starting. Formal ethics approval was not required as the research was classed as a quality improvement project within the trust and approved and signed off on this basis by the then Chief Executive Officer acting on behalf of the Board of Management. Furthermore, patients and members of the public were not involved in the design, reporting, or conduct of the study. Additionally, neither identifiable information nor vulnerable individuals were involved in the study.

### 2.5 Data collection

A theory-generating, semi-structured interview guide explored ‘whether’, ‘how’, and ‘why’ the governance model broke down professional siloes ( Van Audenhove, 2007 ). Each interview lasted an hour, on average. The unique characteristics of the theory-generating interviews are the communicative nature and analytic reconstruction of the subjective dimension of the expert's knowledge, which offer a starting point towards the formulation of the theory ( Bogner and Menz, 2009 ). In our study, the first author (NSB) was allowed to silently observe multiple managerial meetings for one month before conducting the interviews. During these meetings, the first author was able to gain an understanding from an outsider perspective of the phases, changes, obstacles, and enablers the trust navigated over the years.

The interview questions were informed by the knowledge and insight gained during observing meetings and supported by a review of the literature ( Degeling *et al.* , 2003 ; Davies *et al.* , 2003 ; Giordano, 2010 ; Storkholm *et al.* , 2017 ; Dellve *et al.* , 2018 ). We did not have a rigid pre-set theory, instead we leveraged the theory-generating interview approach to explore the experts' insider perspective, and accordingly, formulate a theory based on the themes that stood out the most during the interviews. The questions were formulated by the first and last authors (NSB and BD). The Chief Executive Officer of the trust at the time validated clarity of language and ensured that the regional perspective was sufficiently elicited. Three independent experts reviewed the guide; then, five pilot interviews were conducted to test the interview tool before widespread implementation ( Yin, 2003 ). No changes were made based on the pilot interviews. The qualitative portion of the interview consisted of open-ended questions, during which different themes were explored and specific topics that interviewees mentioned were probed for additional insight. Through the interviews, the history and evolution of the business model were reviewed. All interviews were audio-recorded and fully transcribed by the first author (NSB). Qualitative questions were coupled with verbally administered quantitative questions which were scored on a 10-point scale, with one indicating ‘completely disagree’ and 10 indicating ‘completely agree’. Pooled scale results mirrored sentiments expressed by the interviewees. Quantitative questions were constructed based on findings of previous published research regarding conflicting priorities of different professional groups and the extent to which these groups felt involved in decision making. We aimed to explore whether the new governance model contributed to a shift in the tribalistic perceptions reported by previous research and to match it with the narrative collected in the qualitative portion of the interview.

### 2.6 Data analysis

Qualitative content analysis was conducted and important themes were clustered through thematic coding ( Gibbs, 2007 ). For the quantitative data, descriptive statistics were conducted by pooling the results and calculating the average value for the whole sample and for each professional group.

## 3. Results

**Figure 1:**
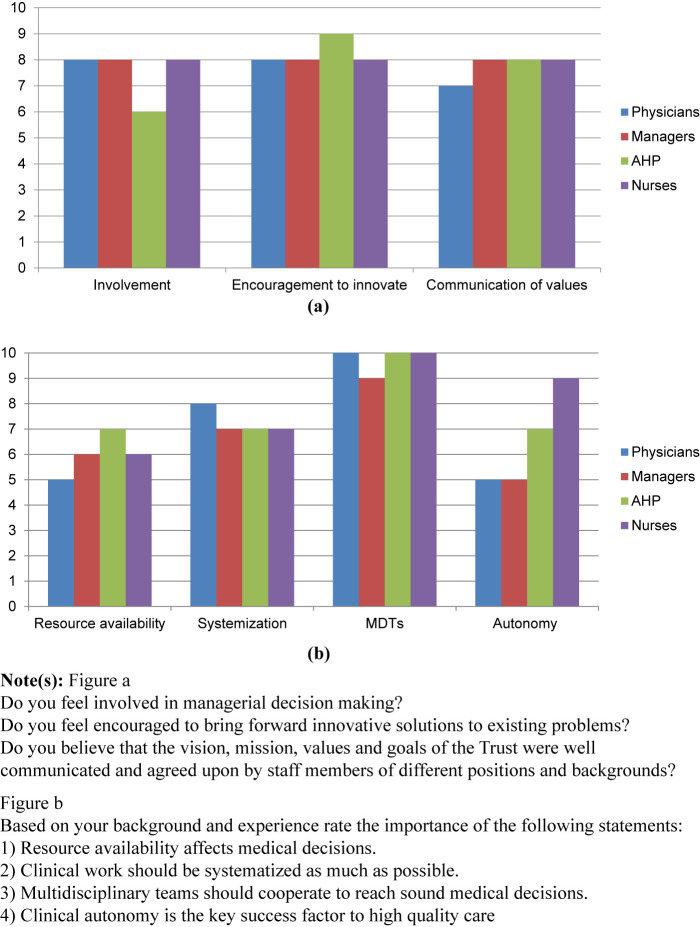
Scorings and ratings of different professional groups' perspectives and priorities

Altogether, 27 in-person expert interviews were conducted (nine physicians, eight lay managers, five nurses, and five AHPs). Table 1 lists all participants and their positions in the trust. Mean length of employment in the trust at the time of interview was 17 years. The interview transcripts were analyzed, and the following trends and themes were observed. Answers to scale questions supported interviewees' statements ( Figure 1 ). Five main themes were highlighted by the interviewees during the interview.

### 3.1 Business units governance model

Interviewees explained that a managerial restructure has been evolving since the early 2000s. Under the new structure, the focus for the operational development were five business units comprising: Child Health, Surgery, Medicine, Clinical Support Services, and Community Services. All units operated on a bipartite system, led by a clinical director and a general manager (Deputy Director level) who had equal power. As interviewees described, business units were substantially autonomous; each business unit negotiated and managed its own service contracts, operational strategy, performance delivery, quality standards, financial targets, and outcome evaluation: *“The business unit model … is having a managerial [personnel] and clinician linked very closely together. [The benefits were] not only … its powers of delegation and its autonomy, but also having alignment between clinical and non-clinical workforces and that in itself being cascaded through the business unit structure. I think that has been really innovative. We are reaping the rewards of that now” (Manager).*

Different professional groups highlighted several advantages to this managerial approach. Physicians noted that the new model helped clinical teams develop a sense of ownership of the trust's finances: *“Our clinically led system has worked well for us … giving clinical and financial control but also accountability and responsibility to clinical teams really engages people. It's not some distant system that is funding them, it's their money, they are managing it within that clinical service” (* Physician). It also helped clinicians gain leadership skills: *“Developing the skills, motivation, and abilities of your clinicians as leaders … we have our clinicians doing 50% of the time clinical stuff, and 50% managerial stuff paired with a senior manager”* (Physician). Moreover, including physicians in top leadership positions led to better reception among the rest of the medical staff, and participants described how this improved patient outcomes: *“It [the business units] helped us drive forward some of the infection control agenda, because if the cascading is coming from clinicians down to clinicians that works better than if it comes from managers down to clinicians”* (Physician).

Nurses and AHPs had similar views; both groups highlighted that the business unit structure empowered clinicians: *“The system very much gives clinical staff a voice in the organization so things like the heads of departments … whether they would be nurses or medics, have a very combined, blended approach with the management team”* (Nurse). They also indicated that it allowed harmonization between professional groups: *“I think it's great to have that relationship between clinicians and managers working together, because managers are seeing from one perspective and clinicians are seeing from another. By marrying them both together, then you can actually work through and come to an agreement … Obviously, everybody is working for the benefit of the patient, but people look at it from different angles”* (AHP).

Managers stated that this managerial approach ensured that cost saving strategies adopted by the trust were not imposed on the clinical teams in a hierarchal manner: *“Whatever cost improvements we come up with, they need to be clinically driven, so that's why the business unit structure … works really well”* (Manager).

To explore whether the business units' governance model and the pairing of managers and clinicians has affected the way different professional groups perceived the importance of budget constraints, interviewees were asked to score the importance of resource availability on their decision making on a scale of 1–10. Figure 1 shows average reported results for different questions. Unlike previous research which showed discrepancy in different professional groups' attitudes, with lay managers usually prioritizing monetary considerations far more than clinicians, in our cohort, physicians, managers, AHPs, and nurses scored this item as 5/10, 6/10, 7/10 and 6/10, respectively. Additionally, interviewees were asked to score the importance of working in an integrated multidisciplinary team. This question was scored the highest across the board with a value of 9/10 for managers and 10/10 for all other groups.

### 3.2 Empowering supportive healthcare professions

Interviewees highlighted that over the years, several professions – including nurse practitioners, nurse specialists, pharmacists, physiotherapists, radiologists, mid-wives – that were initially typically perceived as supportive workforce to physicians, were gradually given enhanced powers and their roles were expanded to allow them to perform on a more autonomous basis: *“We developed our workforce [in a manner] not typical for the NHS, expanding roles beyond the traditional boundaries. Nurse practitioners, specialist nurses, prescribing pharmacists working in clinical areas and a whole range of people moving … into new areas that were previously seen as the domains of other professions”* (Physician).

Empowering them in their fields relieved physicians' pressure, enabling those professionals to excel and improving the standard of care. Nurse practitioners and nurse specialists became the driving force in non-emergency settings. *“It has probably been developed over the last ten years, there was a huge investment made in training nursing staff into a more advanced level. We have nurse practitioners that cover the wards, and they are basically the doctor for that ward. We have emergency nurse practitioners that cover our ED [emergency department], so they see, treat and discharge patients, patients will never see a doctor. We also have specialist nurses who run clinics and have taken over things like endoscopy lists for routine low-risk cases* ” (Nurse).

Similar to nurses, other groups including clinical pharmacists, radiographers, physiotherapists, nutritionists, and midwives all assumed newly structured roles. *“From 2004 we had all of our base site wards [sub-acute base hospital/UTCs wards] at night managed by night nurse practitioner, nurses who've been given training so that they now function to the level of what would be a foundation year two doctor … Very competent workforce. We also invested heavily 12 years ago into senior clinical pharmacists, who are now all independent prescribers … Huge improvement in our medication error rate because medicines are managed by medicines professionals rather than junior doctors”* (Physician).

Because previous research indicated that nurses and AHPs usually valued systematization of work processes more than other professional groups, we wanted to investigate whether the reformed roles impacted their perspective. Our results showed that physicians scored systemization of work processes as 8/10, the highest reported score among all groups, while all other groups scored it as 7/10 ( Figure 1 ).

### 3.3 Intensifying consultants' coverage

Interviewees indicated that given the clinical benefits that had been identified by early intervention of a consultant in a patient's treatment pathway, it was essential to expand consultants' presence beyond the traditional nine a.m. to five p.m. weekdays schedule: *“We have to accept that the consultants are the most senior doctors with the most experience and that was the main drive for change [opening NSECH]”* (Nurse). Moreover, the hands-off nature of the consultants' workforce was gradually shifted to a more active one: “ *The traditional model of consultant working in this country is that most of the time the show is run by junior doctors and the consultant swings in to do his ward round and his clinic but is pretty hands-off. We recognized back in 2003–2004 we had to say to the consultant actually you do not swing by and do the ward round, you are physically present for the full shift” (* Physician).

Consequently, this gradual shift allowed enough time to perfect the model before moving into the new hospital: *“We changed the clinical model in 2004. We moved to having acute care physicians working in the very front of house, all of us started working extended working days. We started to introduce the night nurse practitioners for the base wards, and we started 7 days working by consultant. From 2004–2015 we had that way of working so we refined and tweaked it”* (Physician). This in turn enabled around-the-clock consultant coverage from the first day NSECH was opened in 2015, improving patients' safety due to the consultants' presence as well as approachability: *“Consultants are now seen by members of the staff and patients more as human beings. I think they are more approachable, which … makes in my opinion a safer environment, because people are now not frightened to approach a consultant with the problem”* (Nurse).

For the quantitative portion, our findings were similar to the previous two themes. Unlike previous research which indicated that physicians usually felt very strongly about their autonomy, physicians in our cohort scored autonomy with a value of 5/10, like managers. While AHPs and nurses scored it as 7/10 and 9/10, respectively ( Figure 1 ).

### 3.4 The idea of establishing NSECH

Interviewees were asked whether the idea of opening a dedicated emergency center could be seen as a ‘top-down’ or ‘bottom-up’ idea. They indicated that because of the overlap between clinicians and managers, and since many clinicians worked half-time in managerial positions, it was challenging to identify whether the idea came from a top-down or bottom-up principle in the conventional definition. Instead, many staff members explained it as not being an economic or management direction, but instead a clinically driven decision. *“Without a doubt this wasn't just a doctor, nurse focus led thing, it was all the clinical staff and a massive process of the organisational change which involved everybody. So, from a leadership and direction point of view, we had senior clinicians doing that with working alongside managers hand in hand. I would say one couldn't work without the other, through integrated working … that involved thousands, literally thousands of staff, who contributed ultimately to that change.”* (Nurse).

For this theme, we asked our interviewees two quantitative questions. We asked them to score their involvement in decision making and to which extent they felt encouraged to innovate. Physicians, managers and nurses had a similar score of 8/10 for both questions. AHPs scored these two questions as 6/10 and 9/10, respectively ( Figure 1 ).

### 3.5 Long-term planning

As highlighted in several previous quotes, the processes of changing the governance model on one hand, and the clinical model on the other hand were thoroughly planned, gradual, stepwise processes. Long-term, meticulous, multidisciplinary planning was an overarching theme encompassing multiple frontiers. Centralizing emergency care into one location was envisioned at least a decade before implementation, with steps towards that goal taken over the years: *“We didn't try to take over the world from day one, it's been an evolution of well over ten years”* (AHP); *“We have been doing steps for the last 10–15 years, it is a stepwise progression rather than an absolute innovation. The first lesson, you don't go from zero to hero in a blink of an eye”* (Physician).

To assess whether different professional groups felt that the overall direction of the trust and its values were communicated clearly throughout the long-term process of change, interviewees were asked to score whether the values, mission and vision of the trust were well communicated. Physicians gave a score of 7/10, while the rest of the groups scored it as 8/10. Finally, the last quantitative question demonstrated that all professional groups believed that there was a correlation between clinician representation in management and the trust's ability to embark upon the clinical model modification and open NSECH. Physicians and nurses scored the correlation 9/10 while managers and AHPs scored it 8/10. Without the business unit structure and clinician representation at a senior management level, interviewees doubt that opening the new emergency hospital would have been possible or successful ( Figure 1 ).

## 4. Discussion

Our findings indicate that healthcare tribalism could be overcome by addressing the historically low representation of clinicians at the senior management level and ensuring the alignment of core values between the different professional groups. Several previous studies have shown that key players within healthcare have discrepant attitudes, leading to professional and cultural conflicts ( Degeling *et al.* , 2003 ; Storkholm *et al.* , 2017 ; Dellve *et al.* , 2018 ). Our results identified an approach to governance which explicitly sought to address these discrepancies by introducing a shared decision model. The answers of interviewees in all four professional groups were closely aligned, both quantitatively and qualitatively, and issues frequently identified as resulting from tribalistic attitudes did not emerge in this study.

For example, unlike previous research, staff did not feel that resource availability (austerity impact) had adversely affected clinical performance; staff were supportive of work process systematization; multi-disciplinary team working was overwhelmingly supported; and physicians did not rate their autonomy as highly important, indicating that they were willingly sharing it with other professional groups. Interestingly, although managers and physicians gave the same score, nurses and AHPs ranked the importance of their autonomy higher than physicians did. The close linkage between physicians and managers may have made their views similar; conversely, nurses and AHPs who may not have had much autonomy historically were now celebrating their autonomy and valuing it higher than others. Intriguingly, physicians and managers had the closest alignment on most answers, suggesting that the business unit structure gave them the opportunity to overcome the barriers and misconceptions of professional tribalism.

The managerial and governance structure of the NHS witnessed a drastic makeover about forty years ago. The report chaired by Roy Griffiths in 1983 facilitated the transition from the era of ‘consensus management’ to one of ‘general management’ ( Lewis, 2014 ). In the former, multiple parties had to agree to all decisions, which could have led to stalemate and individuals protecting their own interest rather than the institution's. Griffiths stated that if Florence Nightingale was to carry her lamp through the corridors of the NHS, she would not be able to find the person in charge. Accordingly, the report advocated for appointing a general manager at every level of the NHS and an NHS management board. These newly created posts were to be filled by the best person for the job, Griffiths recommended.

Several unintended consequences followed this transformation. Two thirds of the new roles were filled by an existing administrator who was typically viewed as the most suitable person for the job. Although Griffiths' recommendations were to involve clinicians more closely in the management process, in reality, clinicians were outnumbered and often felt out of place in managerial settings which emphasized finances over care and increased politicization of the NHS as a by-product of the managerial approach ( Morgan, 2014 ). This was reflected in the NHS longitudinal data from the 2000s which suggested that board cultures shifted away from organizational cohesion to prioritizing rules-based achievement and external competitiveness ( Davies and Mannion, 2013 ). During this period, NHCT followed a very different approach to addressing its own external pressures. Overall, the unintended consequences of Griffiths' recommendations might have led to a shift from one extreme to the other and contributed to the emergence of tribalism phenomenon within the NHS. However, NHCT provides a successful example of a balanced middle point on the managerial spectrum. Providing clinical staff with a meaningful voice and role in the management of the organization, while being paired with non-clinical managers, aided in reaching a unified vision and unlocking hidden talents among several other advantages.

Over the past few decades, tribalism and a focus on financial targets were identified as causes of serious healthcare failures in many instances in the NHS, the hardships faced by four trusts were summarized by Goodwin (2019) . The Bristol Royal Infirmary inquiry revealed the existence of an ‘exclusive club’ in which decision-making power was monopolized by the loyal long-term employees entrusted by the Chief Executive. Additionally, different disciplines were nested into strictly siloed groups rather than multidisciplinary teams which meant that managers were not allowed to interfere in clinical decision-making, but also clinicians had no clear path to raise concerns if they were not part of the ‘club’ ( Kennedy, 2001 ). At Mid Staffordshire, the trust was mainly incentivized by financial targets and achieving Foundation trust status. This in turn led to a tolerance of poor standards and disengagement from managerial decision making (even at a consultant level) due to fear of repercussion ( Francis, 2013 ). Similarly, the Liverpool Community Health trust was also driven by finances and the ambition of attaining the Foundation trust status. Consequently, a bullying leadership culture developed resulting in understaffing ( Kirkup, 2018 ). The Morecambe Bay trust exhibited a significant degree of tribalism between different professional groups. The lack of willingness to collaborate endangered patients' safety and well-being ( CBE, 2015 ). In contrast, NHCT did not let the financial pressure dictate its goals. On the contrary, it invested in training healthcare staff to reach their ultimate professional capability and in constructing and furnishing a state-of-the-art new hospital, all to enable the trust to deliver high quality care.

Goodwin concluded that addressing the circumstances that lead to tribalism, one at a time, is the way forward, yet acknowledging that visible shifts can take many years due to the multifactorial influences that come into play such as the day-to-day practices, guidelines set by professional bodies, and the wider social conditions like policy context and societal expectations ( Goodwin, 2019 ). These findings were reinforced by Hunter *et al.* who investigated the main impediments faced by the regional initiative ‘The North East Transformation System’ ( Hunter *et al.* , 2015 ). The authors stress that changing the culture is a ‘never-ending journey’ that cannot be rushed. However, unfortunately, such an approach is often challenged in a public service context like the NHS which demands quick, and tangible results and which itself does not have the stable political atmosphere to allow long lead times given to establishing novel ways of working and the patience allowed for them to produce results ( Hunter *et al.* , 2015 ). Nevertheless, NHCT managed to pursue a model it envisioned more than a decade in advance. With a clear leadership message and driven by a desire to focus on quality of care, the trust was able to create a unique culture that enabled it to withstand political changes over the years.

Tribalism stemmed from several reasons, as we highlighted in the introduction these reasons include historic gender-restricted roles, educational approach, and psychological barriers. Consequently, respect and understanding the responsibilities of different professional roles have been described as problematic issues in the healthcare context ( Ebert *et al.* , 2014 ). Ebert *et al.* concluded that nurses believed physicians were clueless about nurses' responsibilities, they did not trust their examinations and treated them with disrespect. Nurses, on the other hand, referred to physicians as ‘elite’ and worthy of respect. The authors also found that, pharmacists had the impression that physicians perceived them as ‘wannabe’ doctors and often undermined their role. This was supported by nurses' statement *“They [pharmacists] will never actually confront a doctor”, “it always ends up that the doctor has the final say”* (Nurse) ( Ebert *et al.* , 2014 ). These challenges did not occur in NHCT due to the intertwining of several factors which culminated in the harmonization of different professional groups. A key factor was the active effort the trust invested in enabling each professional to reach their maximum capability. Physicians in our study referred to nurses as a very competent workforce that can function at the level of a second-year foundation doctor and described pharmacists as medication experts who drove down the medication error rate. Moreover, nurses in NHCT perceived consultants as more approachable, in their own words ‘consultants are seen like human beings’. This demonstrated that NHCT successfully abolished the rigid hierarchical segregation. All of this was reinforced by the governance model which allowed different professions to be fairly represented.

A recent Harvard Business Review report listed four approaches to mitigating professional tribalism ( Kovach, 2017 ), all of which were successfully used by NHCT. First, ‘Managing the Psychology of the Employees’, NHCT successfully created a unique work culture in which employees felt involved in decision making, encouraged to innovate, and certain that the values of the organization were communicated properly. Second, ‘Breaking Down Silos’, this was successfully implemented through the business unit structure. The equivalent authority of a clinician and a manager allowed both groups to communicate, to moderate their perspectives, to compromise, and to agree on the best way forward for patients' benefit. Third, ‘Managing Executive Egos’ —that is, ensuring senior leadership is sending the right message—was directly cited by one of the interviewees who mentioned that the high degree of collaboration and mutual respect between the clinical and non-clinical teams has been cascaded throughout the different levels of the organization. Fourth, ‘Reframing’, indicating the leader's responsibility to frame the environment for their followers, was again demonstrated through the ownership of the operational and financial strategy of the trust by the bipartite teams managing the business units. This was a consequence of having proper representation of clinicians on managerial teams and the point interviewees highlighted regarding clinical orders being cascaded better when clinicians are addressing other clinicians rather than lay managers addressing clinicians.

This study has several potential limitations. First, like any case study, the results cannot be generalized on a wide scale and are specific to the case at hand. Nonetheless, we outline a range of managerial approaches that are applicable and could add value to diverse settings not limited to this particular case study. Second, this study was conducted several years ago; however, we believe that our findings are even more relevant and insightful now given the global pandemic and the steep inflation which has exerted immense pressure on healthcare systems worldwide. These pressures compounded with another impending financial crisis, warrant reinventing the managerial approach to increase efficiency and reduce waste ( Moynihan *et al.* , 2020 ). To achieve this, the governance model plays a vital role in bridging gaps between professional groups, to reach a unified vision. Our research describes a governance model that achieved these goals. Moreover, different challenges for which we propose solutions in this study, like the professional tribalism, the ‘weekend effect’ and the poor representation of clinicians on the senior management team, all remain persistent issues that recent publications are still trying to resolve. Third, the study was conducted in May–August 2016, exactly one year after opening the NSECH. Despite this short period, our findings are not limited to the events that followed the launch of the new hospital; we retrospectively investigate the factors that enabled this change to happen in the first place. Fourth, since most of the interviewees held managerial positions and were involved in the planning and establishment of the NSECH, their answers to the scale questions could be perceived as biased. This limitation was mitigated by using interviewees' statements collected during the qualitative portion of the interview as well as the CQC assessment to support the quantitative data. Additionally, at least one interviewee in each of the four categories did not hold a managerial position. Finally, some of the events that interviewees referred to in their statements date back to the early 2000s and span over a long period of time, which could pose the risk of recall bias. Nonetheless, this long period was particularly important to highlight that the achieved success was a result of the long lead times given to establishing novel ways of working and the patience needed for it to produce results. In summary, this study offers an insightful narrative of over a decade-long effort, culminating in a clinician-led governance model and an innovative clinical model restructure.

In conclusion, this case illustrates the potential of healthcare organizations once they overcome professional tribalism. Given the long-standing strains on healthcare systems worldwide, hospitals, policy makers and funding bodies can leverage our findings for inspiration and guidance in developing and implementing innovative governance models and restructuring of clinical models.

## Figures and Tables

**Table 1:** List of interviewees

Job title at the time of the interview	Background	Years in the trust	Managerial duties	Supervisees
Chief Executive of the Trust	Physician	28	Yes	9,300 Employees
Divisional Medical Director and Consultant in Geriatrics and General Medicine	Physician	22	Yes	9,000 Employees
Executive Medical Director and Consultant in Intensive Care and Anaesthesia	Physician	18	Yes	300 Consultants
Deputy Medical Director, Caldicott Guardian and Consultant in Respiratory Medicine	Physician	18	Yes	350 Physicians
Business Unit Director and Consultant Cardiologist	Physician	21	Yes	2,000 Employees
Clinical Director of Orthopaedics and Consultant Orthopaedic Surgeon	Physician	17	Yes	24 Orthopaedic Surgeons
Director of Infection, Prevention and Control and Consultant Medical Microbiologist	Physician	12	Yes	16 Microbiologists
Director of Education and Consultant in Haematology	Physician	15	Yes	12 Physicians
Foundation Year 2 Doctor	Physician	2	No	None
Deputy Chief Executive and Executive Director of Operations	Manager	20	Yes	5,000 Employees
Deputy Chief Executive and Executive of Performance and Governance	Manager	13	Yes	50 Employees
Human Resources Manager	Manager	13	Yes	5 Employees
Human Resources Business Partner	Manager	7	Yes	5 Employees
Deputy Director of Finance	Manager	26	Yes	120 Employees
Chief of Finance Manager	Manager	15	Yes	6 Employees
General Manager	Manager	20	Supportive role	None
Operational Service Manager in Medicine Business Unit	Manager	16	Yes	240 Employees
Chief Pharmacist and Clinical Director for Medicines Optimization	Allied health professional	14	Yes	175 Pharmacists
Specialist Clinical Pharmacist	Allied health professional	2	No	None
Head of Midwifery	Allied health professional	32	Yes	120 Midwives
Radiographer Lead at NSECH	Allied health professional	28	Yes	50 Radiographers
Physiotherapist	Allied health professional	22	Yes	30 Physiotherapists
Interim Executive Director of Nursing	Nurse	16	Yes	3,000 Nurses and 500 allied health professionals
Interim Deputy Director of Nursing	Nurse	18	Yes	10 Nurses
Upper Gastrointestinal Clinical Nurse Specialist and Nurse Endoscopist	Nurse	17	Yes	7 Nurses
Assistant Endoscopist	Nurse	15	No	None
Senior Staff Nurse and Junior Emergency Care Nurse Practitioner	Nurse	16	Yes	5 Nurses
